# Effects of UV-B radiation on leaf hair traits of invasive plants—Combining historical herbarium records with novel remote sensing data

**DOI:** 10.1371/journal.pone.0175671

**Published:** 2017-04-17

**Authors:** Tomáš Václavík, Michael Beckmann, Anna F. Cord, Anja M. Bindewald

**Affiliations:** 1 UFZ–Helmholtz Centre for Environmental Research, Department of Computational Landscape Ecology, Leipzig, Germany; 2 Palacký University Olomouc, Department of Ecology and Environmental Sciences, Faculty of Science, Olomouc, Czech Republic; 3 University of Applied Sciences Bremen, Bremen, Germany; College of Agricultural Sciences, UNITED STATES

## Abstract

Ultraviolet-B (UV-B) radiation is a key but under-researched environmental factor that initiates diverse responses in plants, potentially affecting their distribution. To date, only a few macroecological studies have examined adaptations of plant species to different levels of UV-B. Here, we combined herbarium specimens of *Hieracium pilosella* L. and *Echium vulgare* L. with a novel UV-B dataset to examine differences in leaf hair traits between the plants’ native and alien ranges. We analysed scans of 336 herbarium specimens using standardized measurements of leaf area, hair density (both species) and hair length (*H*. *pilosella* only). While accounting for other bioclimatic variables (i.e. temperature, precipitation) and effects of herbivory, we examined whether UV-B exposure explains the variability and geographical distribution of these traits in the native (Northern Hemisphere) vs. the alien (Southern Hemisphere) range. UV-B explained the largest proportion of the variability and geographical distribution of hair length in *H*. *pilosella* (relative influence 67.1%), and hair density in *E*. *vulgare* (66.2%). Corresponding with higher UV-B, foliar hairs were 25% longer for *H*. *pilosella* and 25% denser for *E*. *vulgare* in records from the Southern as compared to those from the Northern Hemisphere. However, focusing on each hemisphere separately or controlling for its effect in a regression analysis, we found no apparent influence of UV-B radiation on hair traits. Thus, our findings did not confirm previous experimental studies which suggested that foliar hairs may respond to higher UV-B intensities, presumably offering protection against detrimental levels of radiation. We cannot rule out UV-B radiation as a possible driver because UV-B radiation was the only considered variable that differed substantially between the hemispheres, while bioclimatic conditions (e.g. temperature, precipitation) and other considered variables (herbivory damage, collection date) were at similar levels. However, given that either non-significant or inconclusive relationships were detected within hemispheres, alternative explanations of the differences in foliar hairs are more likely, including the effects of environment, genotypes or herbivory.

## Introduction

Ultraviolet-B radiation (UV-B, wavelengths of 280–315 nm) is a key environmental factor that initiates diverse responses at numerous levels of plant performance and in a wide range of plant species [1, 2]. For example, UV-B radiation increases the concentration of UV-absorbing compounds in plant tissues, reduces biomass production and photosynthetic activity (e.g. [3, 4]) and causes DNA and protein damage [5, 6]. In response to UV-B exposure, plant species have developed a diverse set of mechanisms to counteract the imposed stress [1], for example by changing leaf morphology with increased epidermal and epicuticular thickness and reduced leaf area (as reviewed in [7, 8, 9]). An important role in the protection of leaf tissues is also attributed to foliar hairs [10]. For various species, studies have detected that UV-B exposure increases foliar hair density [8, 11–13] or hair length [14]. These responses have been generally linked to increased absorbance and reflection of harmful UV-B because even if hairs cover only a fraction of the leaf area, they may provide a cumulative shading effect due to the changing angle of incoming radiation [14]. Thus, our knowledge on the specific effects of UV-B radiation on plant morphology and physiology is growing but more biogeographical research is needed to better understand the role of UV-B radiation as a broad-scale environmental driver and its potential impact on the distribution patterns of UV-B sensitive plant species.

The intensity of UV-B radiation varies spatially across the globe, following complex latitudinal [15], altitudinal and temporal patterns [16]. For example, the well-known hemispheric difference in UV-B exposures is partly attributed to the decreased levels of atmospheric ozone over Antarctica and Australia [17] and to the inclination of the Earth’s orbit that leads to a smaller distance between the Earth and the sun during the austral summer [16, 18]. In addition, the intensities in UV-B radiation have changed over time, increasing in some regions while decreasing in others [19], particularly in the past few decades. Recently, the profound effects of higher UV-B radiation intensities on ecosystems, especially in the Southern Hemisphere, have received growing attention [20].

The majority of our knowledge of plants’ response mechanisms to the stress caused by UV-B is based on comparatively local, short-term, experimental studies, often set up in growth chambers or using supplemental lighting/filtering in the field (e.g. [21, 22] but see [23]). However, alien plant species provide opportunities to investigate effects of different environmental conditions across larger geographic and temporal scales. Given the substantial number of plant species spreading into new regions with new sets of environmental conditions, biological invasions may be viewed as ‘natural experiments’ [24] that allow us to investigate responses in leaf morphology to different UV-B intensities. This can be done, for example, by comparing alien plant populations of the Southern Hemisphere with their native populations from similar latitudes and climatic conditions in the Northern Hemisphere.

Only a few studies so far have investigated UV-B adaptations of plant species in their native and alien regions (but see [8, 14, 25]), probably due to the scarcity of long-term species records and the previous lack of large-scale spatial data on UV-B radiation. However, global UV-B radiation climatologies have been recently developed (glUV [26]) which summarize remotely sensed measurements of UV-B radiation into six ecologically meaningful UV-B variables at a 15-arc minute spatial resolution. These data provide the opportunity to study the effects of UV-B radiation on plant morphology during invasion and to address the role of UV-B as an environmental factor that links functional response traits of plant species to their distribution patterns.

In addition, using historical species records to address biogeographical questions at large spatial scales has proven to provide valuable insights. For example, herbarium specimens have been used to examine changes in plant size [27] and leaf morphology in response to climate change and atmospheric CO_2_ increases [28, 29]. Furthermore, the vulnerability of habitats to plant invasions [30] and the evolution of plant sizes on Southern Pacific islands [31] have recently been analyzed with the help of herbarium records. To our knowledge, changes in leaf hair traits in response to different patterns of UV-B exposure have not yet been investigated using herbarium records.

Here, we examined changes in two leaf-hair traits (hair length and hair density) in response to different levels of UV-B radiation by combining historical herbarium records with a global UV-B dataset (glUV). In contrast to previous experimental studies that have investigated the response of plant traits to UV-B exposure under artificial growing conditions, we conducted an observational study using specimens collected under natural growing conditions. We examined *Hieracium pilosella* L. agg. and *Echium vulgare* L., two plant species native to Europe but invasive in many regions in the Southern Hemisphere, especially New Zealand [32, 33], where they are exposed to comparable climatic conditions but different levels of UV-B irradiance. There is experimental evidence that UV-B increases the length of foliar hairs for *H*. *pilosella*, while it does not affect hair density in the same species [14]. For *E*.*vulgare*, it has been shown in an experimental setup that UV-B exposure increases the density of foliar hairs [34]. However, since measuring hair length of *E*. *vulgare*, especially on dried material, is not feasible as the hairs break very easily, we chose to investigate hair density in both species and hair length in *H*. *pilosella* only.

Comparing these leaf hair traits (i.e. hair density and length) as well as traits generally related to leaf morphology (i.e. leaf area and number of hairs per leaf) between disjunct geographical ranges represents a suitable setting to study the macroecological influence of UV-B radiation. Although an observational study based on samples collected under natural growing conditions cannot disentangle the effects of possible genetic changes versus phenotypic plasticity without including information on the spatial distribution of different genotypes, it can show whether the geographic patterns of leaf hair traits correspond with patterns of UV-B irradiation. Here, we specifically ask (1) whether varying levels of UV-B exposure explain the variability and geographical distribution of these two leaf hair traits, and (2) whether significant differences in functional UV-B responses in hair traits exist between specimens collected in the Northern vs. the Southern Hemisphere. We predict that, in comparison with other climatic factors such as precipitation and temperature, UV-B measurements provide the most explanatory value for the distribution of leaf hair phenotypes. Further, we predict that—in accordance with the previously observed patterns—*H*. *pilosella* shows longer foliar hairs while having similar hair densities and *E*. *vulgare* shows denser foliar hairs in specimens collected in the Southern Hemisphere. Assuming that plants reacted plastically in hair traits, phenotypic plasticity of both study species should also be detectable by a positive correlation of hair traits and UV-B levels in each hemisphere separately.

## Materials and methods

### Study species

*Hieracium pilosella* L. (Mouse-ear hawkweed) is a perennial plant species commonly growing in dry grasslands, heathers and open forests in its native range [35] (see Table 1 for overview). In New Zealand, it has become one of the most abundant species in the areas with moderate to low rainfall in the South Island [36]. *Echium vulgare* L. (Blueweed or Viper´s bugloss) is a biennial or perennial plant species typically growing in dry to moderately wet rocky meadows and sandy grasslands in its native range [35] (Table 1). In New Zealand, it is common along roadsides and other open disturbed areas, particularly in drier parts of the South Island [33]. It has become a noxious weed, as it invades natural grasslands where it replaces native plants [37]. We chose these two herbs as they are representative of plant species that are sensitive to UV-B radiation and because their leaf hair trait responses to UV-B radiation have been documented in previous experimental studies which detected increased hair length in *H*. *pilosella* and increased hair density in *E*. *vulgare* [14, 34].

**Table 1:** Overview of the study species *Hieracium pilosella* L. and *Echium vulgare* L. and the number of examined herbaria specimens in their native and alien ranges.

	*Hieracium pilosella*	*Echium vulgare*
Family	Asteraceae	Boraginaceae
Native range	Europe, Western Asia [39]	Europe, Western Asia [35]
Alien range	New Zealand, North America, South America [33, 40]	Australia, North America, South America, Japan, Southern Africa [37]
Specimens by herbaria	AK: 19, JE: 69, LZ: 18, CHR: 102	AK: 17, CHR: 50, JE: 55, LZ: 4; P: 2
Sampling locations of specimens by country	AUT: 8, CZE: 6, GBR: 1, DNK: 1, FRA: 5, GER: 40, HUN: 8, ITA: 12, NOR: 1, NZL: 112, ROU: 2, RUS: 1, SWE: 5, CHE: 6	ARG: 1, AUS: 1, BEL:1, CAN: 1, CZE: 1, GBR: 2, FIN: 2, FRA: 2, GER: 48, ITA: 1, NZL: 59, ROU: 2, RUS: 1, ESP: 1, HUN:1, USA: 4
Total number of specimens	208	128
UV-B response of hair traits under laboratory conditions	Foliar hair length increases under UV-B while density is not affected [14]	Hair density on the upper leaf surface increases when treated with UV-B [34]
Hypotheses tested in this study	Hairs are longer on specimens exposed to higher levels of UV-B (i.e. collected in the Southern Hemisphere); hair density shows no differences between hemispheres	Hair density is higher on specimens exposed to higher levels of UV-B (i.e. collected in the Southern Hemisphere)

Abbreviations: Auckland War Memorial Museum Herbarium (AK); ALLAN Herbarium (CHR); Herbarium Hausknecht Jena (JE); Herbarium Universitatis Lipsiensis (LZ); Museum National d' Histoire Naturelle, Paris (P); New Zealand (NZ); Ultraviolet-B radiation (UV-B); Northern Hemisphere (NH); Southern Hemisphere (SH); country codes are given in ISO 3166–1 alpha-3 format. Refer to Fig 1 for a map of sampling locations.

Given the physical isolation of New Zealand, from which the majority of our samples originated, it would be important to know how many introduction events there are likely to have been. If only one or a few, then the founding population may have been small, and the patterns observed may reflect the characteristics of the founders rather than selection of new habitat conditions, including different levels of UV-B radiation. Unfortunately, no information exists about the number of introduction events that occurred in New Zealand. However, the population of *H*. *pilosella* includes variable genotypes that came from different places in the UK and Central/Eastern Europe [38], implying multiple introduction events.

### Data collection from herbarium specimens

**Fig 1:**
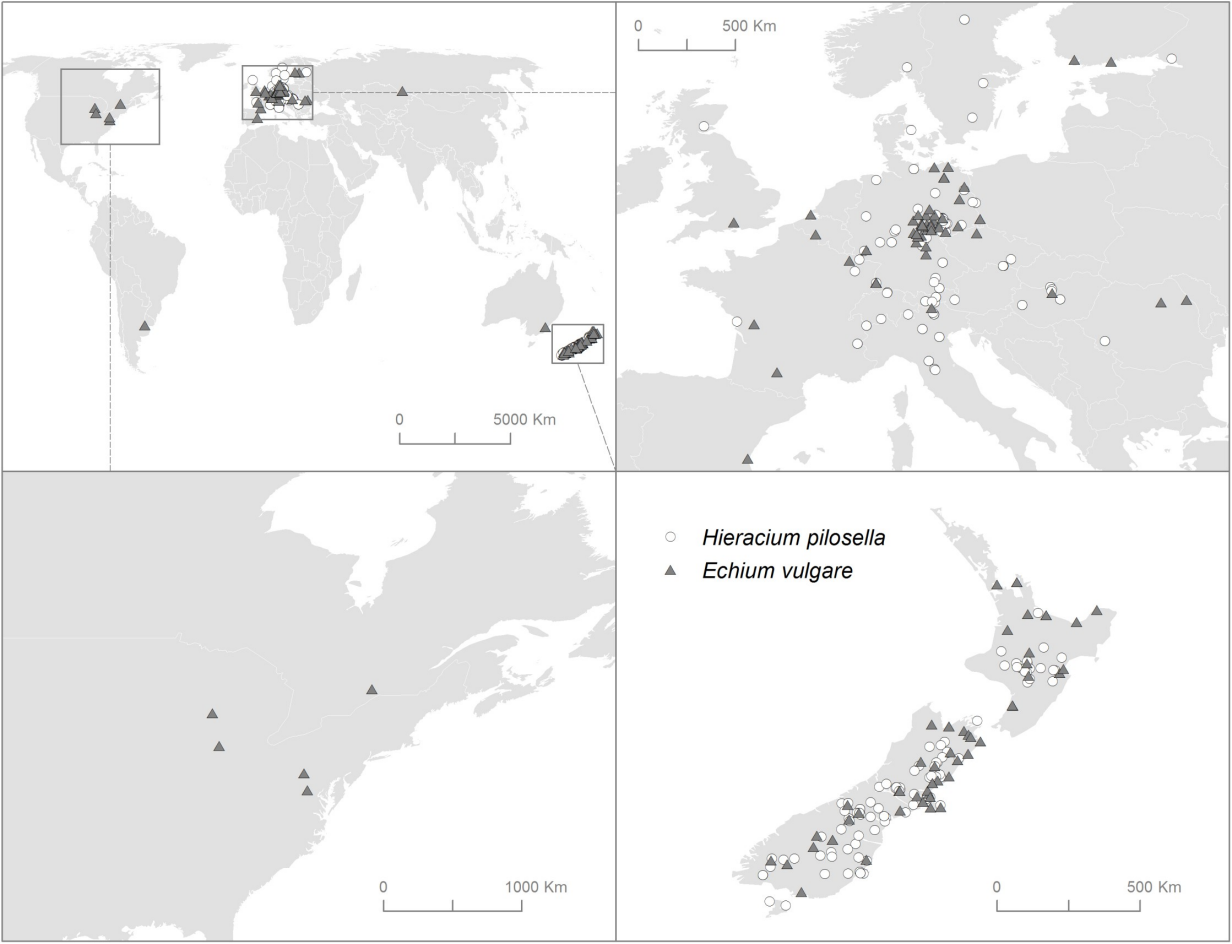
Map of sampling locations of analyzed herbarium specimens.

We investigated leaf hair traits using herbarium specimens collected from five different herbaria (Table 1). We chose herbarium records over fresh samples because herbarium samples allow achieving much wider spatial coverage and longer time span than would be feasible with experimental or field-based approaches. In total, we examined 208 specimens of *H*. *pilosella* (96 from the Northern and 112 from the Southern Hemisphere) and 128 specimens of *E*. *vulgare* (67 from the Northern and 61 from the Southern Hemisphere; Table 1 and Fig 1). The specimens covered a range of UV-B intensities across the entire species’ native range in Europe and a large part of their invasive range, mainly in New Zealand. Therefore, we can consider the included spatial extent to be well representative of the larger geographical ranges of the Northern and Southern Hemispheres. In addition, local measurements quantifying UV-B in New Zealand and Germany at comparable latitude, altitude and meteorological season showed that erythemally weighted irradiance in New Zealand is about 60% higher than in Germany [41]. Such conditions made these regions ideal for our study.

**Fig 2:**
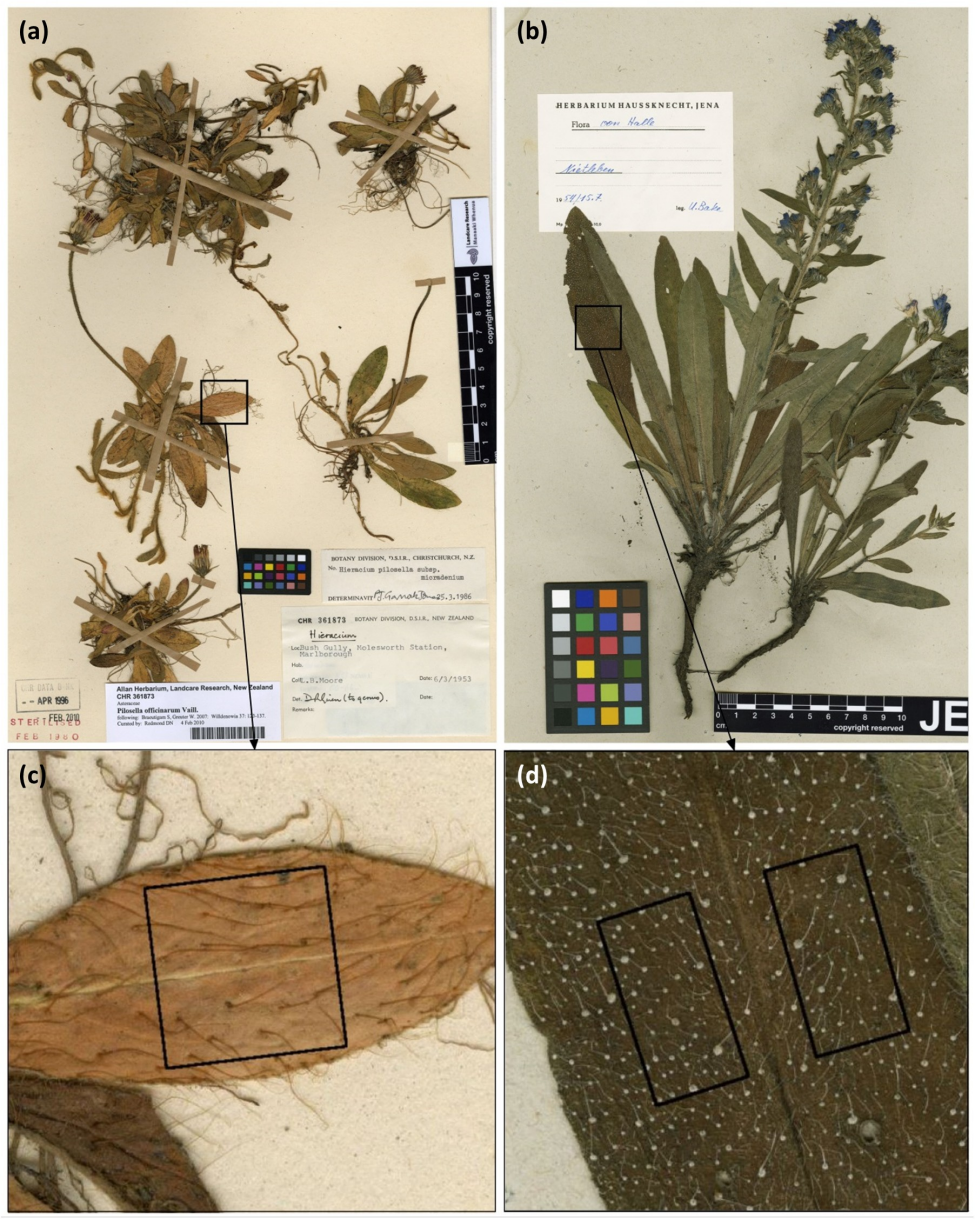
Measurements of herbarium specimens based on digital scans (resolution: 500 dpi). (a) *Hieracium pilosella* L. (here: specimens from ALLAN Herbarium (CHR), New Zealand); (b) *Echium vulgare* L (here: specimens from Herbarium Hausknecht Jena (JE), Germany); (c) Measurements of hair density and length within a 10 x 10 mm square in the middle of the upper leaf surface on a *H*. *pilosella* specimen; (d) Measurement of hair density within two 5 x 10 mm squares placed left and right of the midrib on the upper leaf surface on a *E*. *vulgare* specimen.

We scanned all herbarium specimens with a HerbScan equipped with the Epson Expression 10.000XL scanner that is typically used to digitize specimen collections. The scanner was mounted upside down which enabled scanning without inverting the specimen sheets. Scans were performed with a resolution of 500 dpi that later allowed accurate analyses of hair traits on the upper leaf surfaces (Fig 2). For leaf hair measurements, we chose the largest leaf of the plant’s rosette (or, if not possible, of the stem) on every specimen sheet and measured hair length (*H*. *pilosella*) and density (*E*. *vulgare* and *H*. *pilosella*). Measurements were performed within a 10 x 10 mm square in the middle of the upper leaf surface in the case of *H*. *pilosella* specimens (Fig 2A and 2C) and within two 5 x 10 mm squares positioned left and right of the midrib in the case of *E*. *vulgare* specimens (Fig 2B and 2D). For each sampled leaf per specimen, we recorded the following attributes: leaf area (cm^2^), position of the sample leaf (rosette or stem), hair density (no. of hairs per cm^2^), hair length (mm, *H*. *pilosella* only) on the upper leaf surface, collection date and geographic coordinates of every collection site. If coordinates were not given in decimal degrees we converted them or approximated the location in Google Earth based on the site description in the herbarium record. We did not collect hair length data for *E*. *vulgare* because, unlike in the case of *H*. *pilosella* that has softer and flexible hairs even when dried, hairs of *E*. *vulgare* break easily, so it is impossible to accurately measure their length from herbaria records. All leaf hair measurements were conducted in the GNU Image Manipulation Program (GIMP, Version 2.8.4).

In addition, we estimated the level of herbivory for each individual on which the leaf hair measurements were taken. We only considered herbivory damage that affected fresh leaves, i.e. we ignored feeding damages that apparently happened on the already dried herbarium material. We used a standardized template to visually estimate five different levels of herbivory (0% = intact leaf, 1–5% damage, 6–10%, 11–20%, 21–30%) on the three most damaged leaves per specimen.

### Statistical analyses

Using the geographic coordinates of the specimens, we extracted corresponding values of UV-B radiation and other bioclimatic factors for each specimen to obtain predictors that may explain the variability in leaf hair traits. For UV-B data, we used glUV [42], a global dataset of surface UV-B radiation which represents long-term averages of UV-B conditions at 15-arc minute spatial resolution. glUV is based on daily measurements of NASA’s Ozone Monitoring Instrument (Aura-OMI) satellite mission for the period 2004–2013 and accounts for for possible filtering effects caused by clouds and aerosols. We selected five long-term aggregates of UV-B intensities that are suitable for macroecological analyses as model predictors: Annual Mean UV-B (UVB1), Mean UV-B of Highest Month (UVB3), Mean UV-B of Lowest Month (UVB4), Sum of UV-B Radiation of Highest Quarter (UVB5) and Sum of UV-B Radiation of Lowest Quarter (UVB6). As additional bioclimatic predictors, we derived the following eleven biologically relevant variables from the WorldClim database [43]: Annual Mean Temperature (BIO1), Maximum Temperature of Warmest Month (BIO5), Minimum Temperature of Coldest Month (BIO6), Mean Temperature of Warmest Quarter (BIO10), Mean Temperature of Coldest Quarter (BIO11), Annual Precipitation (BIO12), Precipitation of Wettest Month (BIO13), Precipitation of Driest Month (BIO14), Precipitation of Wettest Quarter (BIO16), Precipitation of Driest Quarter (BIO17) and Altitude. All predictor variables were rescaled to 15-arc minute resolution to match the original resolution of the UV-B data. If several specimens were found within the same 15-arc minute raster cell, the values of the response variables, hair length and density (for *H*. *pilosella*) and hair density (for *E*. *vulgare*), were averaged to avoid pseudo-replication.

We used Boosted Regression Trees (BRT) to examine the degree to which the UV-B and bioclimatic variables explain the variability and geographical distribution of studied leaf traits. BRT (R version 3.0.1 [44]; package ‘gbm’ version 2.1 [45]) is an additive regression model that combines regression trees, a method which repeatedly partitions data with respect to a single explanatory variable using binary splits [46]. BRT includes boosting, i.e. a machine-learning procedure that runs numerous models to improve model explanation and predictive performance. Having a set of collinear variables (S1 Table), we chose the BRT method to identify those predictors that contribute the most to explaining the variability in leaf hair traits. BRT is a suitable method for this purpose because it provides information about the relative importance of predictor variables and because it can handle different types of predictors and interaction effects [47]. The method is robust even for highly correlated variables because when a variable is chosen to make a split in the regression tree, then the redundant variable is less likely to be chosen for a subsequent split. Here, the measure of relative variable influence was based on the number of times a variable was selected for splitting, weighted by the squared improvement to the model as a result of each split, averaged over all trees and scaled to the range of 0 to 100, with higher numbers indicating stronger influence on the response [47]. In order to reduce overfitting, we applied the shrinkage parameter to decrease the influence of single trees on final prediction. We ran 10,000 BRT iterations and selected the most parsimonious model based on the lowest prediction error (root mean square error) calculated from 5-fold cross-validation for each iteration. We fixed the model parameters based on their optimal estimation in the fitting process (as per [46]) and made the model deterministic by using a bag fraction of 1 [45].

The importance of the most relevant variables for the studied hair traits according to the BRT analysis was then further examined using linear regression modelling and t-tests. First, for all samples, we fitted an ordinary least square (OLS) regression model between hair trait variables for both species and those UV-B and bioclimatic variables that were determined by the BRT analysis as having relative influence above zero. In the same models, we also included the collection date and herbivory damage to test for their potential effects. Variables were checked for normality of distribution and log transformed when necessary to improve normality of residuals. We also calculated Cook’s distance to check whether there were single observations in the dataset with large residuals that may have distorted the outcome of the analysis. Models were tested for spatial autocorrelation among regression residuals using Moran’s I statistic, in order to avoid spatial dependence in the error term [48]. Second, we added hemisphere as a categorical variable to the linear model above, running it as an analysis of covariance (ANCOVA). This way we directly tested for an interaction effect between UV-B levels and hemisphere and examined whether the most influential bioclimatic variables were confounded with other potential variables (e.g., shading, nutrients) that may also differ between hemispheres in a consistent fashion. Third, we further examined the most influential variable from each BRT analysis and used scatter plots and t-tests to compare the variance in hair trait data between the respective hemispheres, in order to address the hypothesis that differences in hair traits exist between specimens collected in the Northern vs. the Southern Hemisphere. As leaf hair density may be influenced by the overall leaf size [49], we also calculated Pearson product-moment correlation coefficients for these variables. All statistical analyses were conducted in R 3.0.1 [44].

## Results

**Fig 3:**
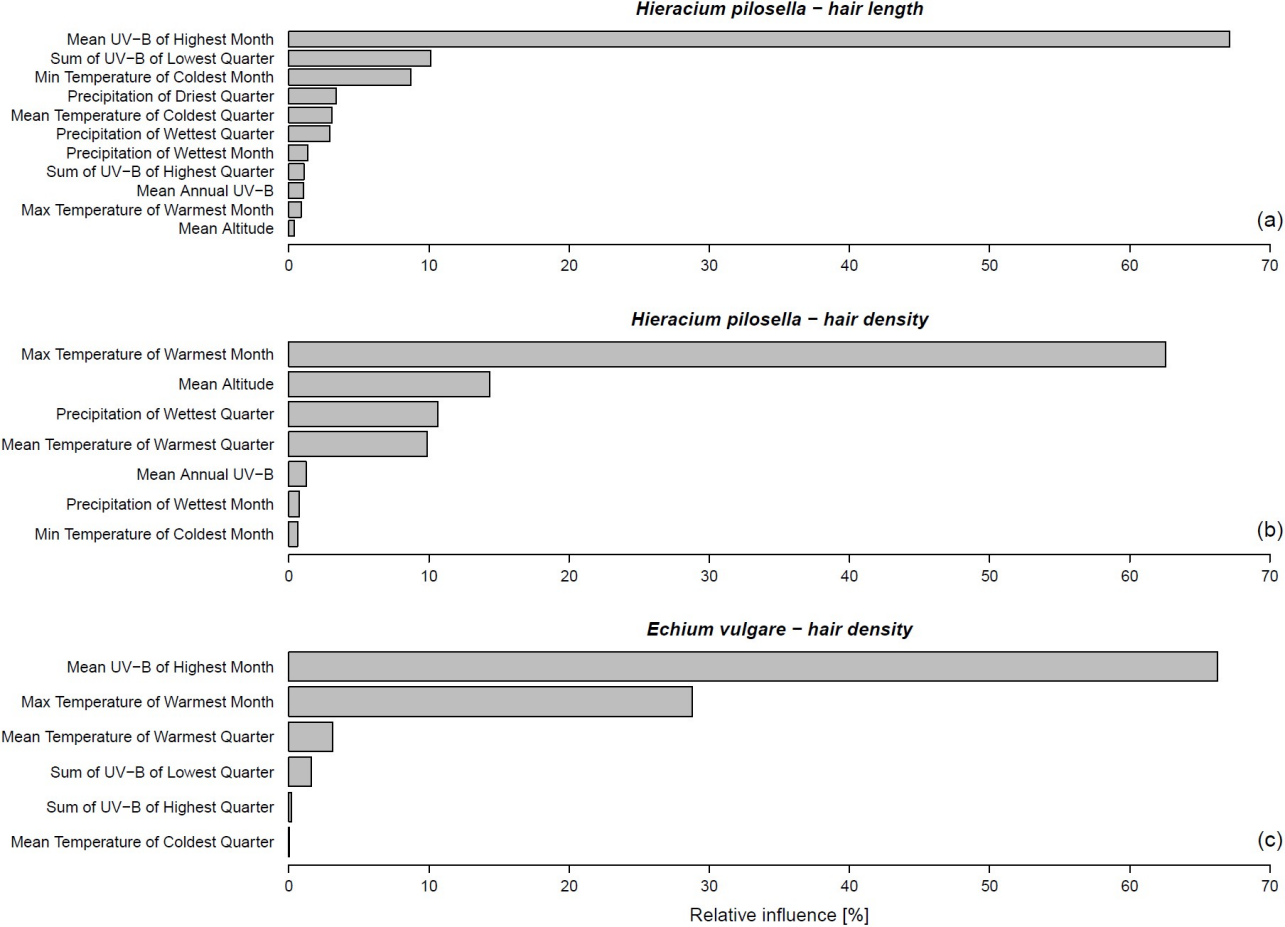
Most important UV-B/bioclimatic variables (predictors) to explain leaf hair trait data (response) based on the Boosted Regression Tree (BRT) analysis. All variables having more than 0% relative influence are shown. UV-B of Highest Month accounts for most variation in hair length for *Hieracium pilosella* (a; rel. influence 67.1%) and leaf hair density for *Echium vulgare* (c; rel. influence 66.2%). Maximum Temperature of Warmest Month accounts for most variation in hair density for *Hieracium pilosella* (b; rel. influence 62.6%). See text for full list of variables used in the BRT analysis.

BRT analysis revealed Mean UV-B of the Highest Month to be the single factor that accounted for most variation in hair length for *H*. *pilosella* (relative influence 67.1%, Fig 3A) and hair density for *E*. *vulgare* (66.2%, Fig 3C). Hair length of *H*. *pilosella* was also partly explained by Sum of UV-B of Lowest Quarter (10.1%) and several other temperature and precipitation variables that each explained less than 10% of variability in the data (Fig 3A). Hair density of *E*. *vulgare* was also partly explained by Maximum Temperature of Warmest Month (28.8%) and several other bioclimatic factors that each contributed less than 5% to model gain (Fig 3C). Hair density of *H*. *pilosella*, however, was mostly explained by Maximum Temperature of Warmest Month (62.6%), followed by Altitude (14.3%) and Precipitation of Wettest Quarter (10.6%, Fig 3B). The only UV-B variable that contributed to model gain was Mean Annual UV-B with 1.2%. BRT analysis of leaf area (S1 Fig) revealed Maximum Temperature of Warmest Month (26.5%) to be accounting for most variation in *H*. *pilosella*, followed by Sum of UV-B of Lowest Quarter (18.0%). Leaf area in *E*. *vulgare* was explained by two variables: Precipitation of Wettest Quarter (71.6%) and Maximum Temperature of Warmest Month (28.4%).

The multiple regression models for those variables with the highest importance according to the BRT analysis and with specimen data from both hemispheres revealed a significant positive effect of Mean UV-B of the Highest Month and Sum of UV-B of Lowest Quarter on the hair length in *H*. *pilosella* (OLS, F(2, 204) = 22.69, R^2^ = 0.18, p<0.001 and p = 0.017, respectively). For *E*. *vulgare*, the model showed a significant positive effect of Mean UV-B of the Highest Month and a negative effect of Maximum Temperature of Warmest Month on hair density (OLS, F(2, 125) = 5.71, R^2^ = 0.08, p = 0.029 and p = 0.043 respectively). Finally, the model for hair density in *H*. *pilosella* revealed a significant positive correlation with Maximum Temperature of Warmest Month, and Altitude (OLS, F(2, 204) = 7.56, R^2^ = 0.07, p = 0.006 and p = 0.027, respectively). When we controlled for the effect of hemisphere directly using ANCOVA, hemisphere substituted the explanatory power of UV-B variables for hair length in *H*. *pilosella* (F(1, 206) = 59.87, R^2^ = 0.23, p<0.001) and hair density in *E*. *vulgare* (F(1, 126) = 13.20, R^2^ = 0.09, p<0.001), explaining approximately the same amount of variance in the data. Hemisphere as a categorical variable was not significant in the model for hair density in *H*. *pilosella* (p = 0.59). No interaction effects were found in any of the models. Tests of spatial autocorrelation among regression residuals were negative for both species (*H*. *pilosella*: Moran’s I = -0.012, p = 0.790, *E*. *vulgare*: Moran’s I = 0.029, p = 0.099) and the calculation of Cook’s distance did not identify any extreme outliers in the dataset, therefore the statistical assumptions of regression analysis were not violated.

**Fig 4:**
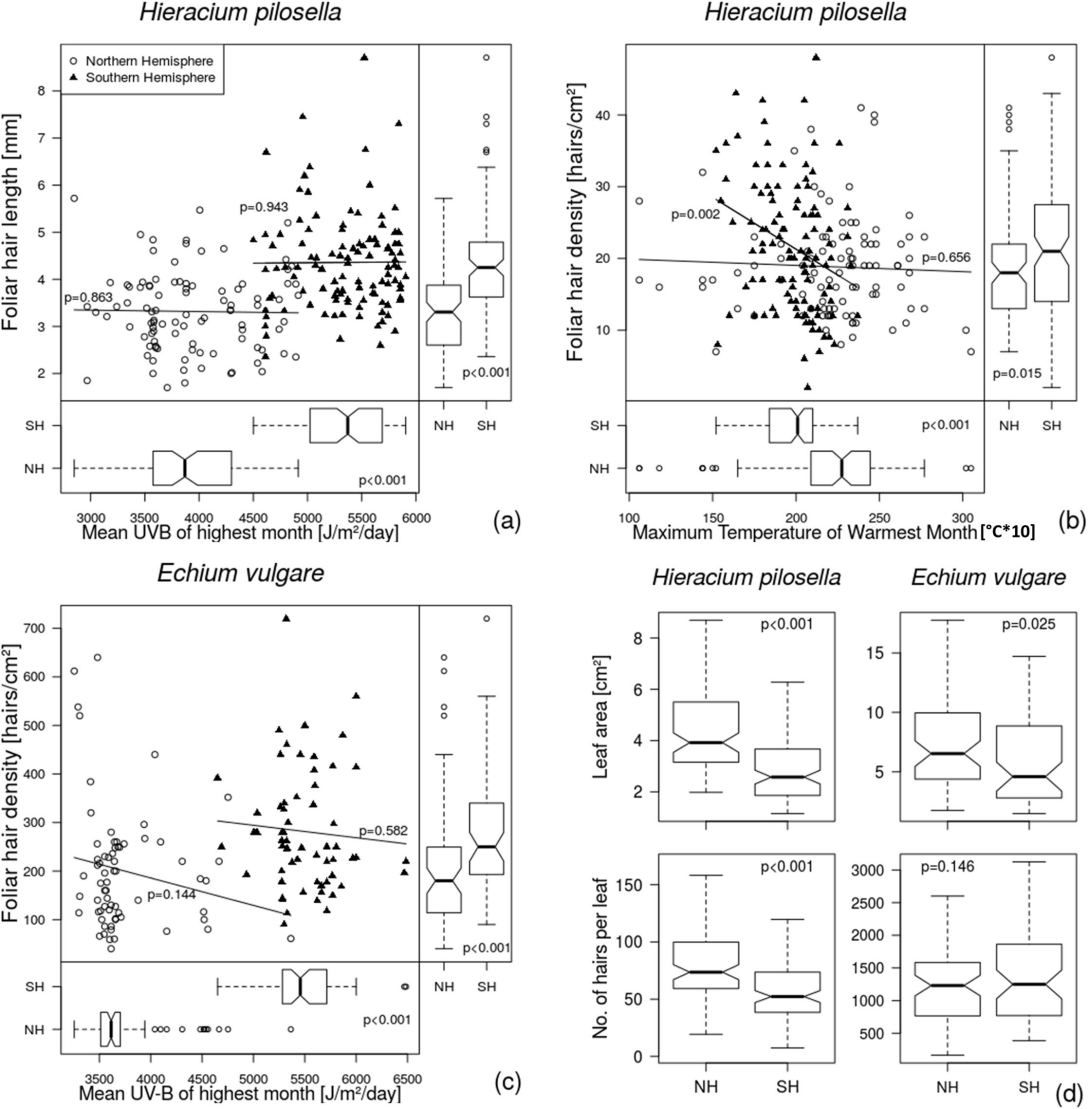
**Relationship between Mean UV-B radiation of Highest Month (a,c) and Maximum Temperatures of the Warmest Month (b) and phenotypic expressions of leaf hair traits represented by (a) foliar hair length, (b) foliar hair density in *Hieracium pilosella* and (c) foliar hair density in *Echium vulgare*.** Records from the Northern Hemisphere are denoted by open circles, records from the Southern Hemisphere are denoted by black triangles. Lines with p-values represent fitted linear regression models. Vertical boxplots with p-values from t-tests show the differences in hair traits between the hemispheres. Horizontal boxplots with p-values from t-tests compare the tested environmental variables between the hemispheres. Panel (d) shows boxplots comparing leaf area [cm^2^] and estimated number of hairs per leaf between specimens collected in the Northern versus the Southern Hemisphere. Abbreviations: NH = Northern Hemisphere, SH = Southern Hemisphere.

Focusing on each hemisphere separately, we found no significant effects of the most influential variables from the BRT analyses (i.e. Mean UV-B of the Highest Month and Maximum Temperature of Warmest Month) on the hair length in *H*. *pilosella* or on the hair density in *E*. *vulgare* in either of the hemispheres (Fig 4A and 4C). Hair density of *H*. *pilosella* even reduced with increasing Mean UV-B of the Highest Month and Maximum Temperature of Warmest Month in the Southern Hemisphere (Fig 4B; S2 Fig). Comparing the herbaria records from different hemispheres showed significant differences in leaf hair traits between the northern and southern specimens (Fig 4A, 4B and 4C, right panels). Leaf hairs of *H*. *pilosella* specimens that were collected in the Southern Hemisphere grew about 25% longer (increase of 1.1 mm, t-test, t = 6.91, p<0.001, Fig 4A) and 15% denser (2.8 more hairs per cm^2^, t-test, t = 2.95, p = 0.015, Fig 4B) than those collected in the Northern Hemisphere. The leaf hair density from *E*. *vulgare* specimens collected in the Southern Hemisphere was about 25% higher compared to those from the Northern Hemisphere (t-test, t = 3.64, p<0.001, Fig 4C) with hair densities reaching more than 700 hairs per cm^2^ for the Southern Hemisphere specimens. These differences in leaf hair traits corresponded with differences in UV-B radiation recorded for the geographic locations of collected specimens. UV-B radiation levels (Mean UV-B of Highest Month) reaching the localities on the Southern Hemisphere were about 30% higher for *H*. *pilosella* specimens and 35% higher for *E*. *vulgare* specimens compared to the Northern Hemisphere (t-test, t = 18.43, p<0.001, Fig 4A and 4C, lower panels).

Herbivory damages were on average lower in the Southern Hemisphere for both *H*. *pilosella* and *E*. *vulgare* (S3 Fig), but this difference between hemispheres was not statistically significant (t-test, t = -1.78, p = 0.076 and t = -1.97, p = 0.051 respectively). The analysis of collection year also revealed no significant relationship between the three tested trait-species combinations and the year the specimens were collected (S4 Fig).

Finally, specimens collected in the Southern Hemisphere displayed reduced leaf area in comparison to the Northern Hemisphere (*H*. *pilosella*: t-test, t = -5.08, p<0.001; *E*. *vulgare*: t-test, t = -3.19, p = 0.025, Fig 4D). Correlation analysis between leaf area and the most influential variables from the BRT analysis for leaf area (i.e. Maximum Temperature of Warmest Month or Precipitation of Wettest Quarter) showed that, while these variables differed significantly between hemispheres, within-hemisphere correlations did not exist (S5 Fig). The total number of hairs per leaf was lower in the Southern Hemisphere only for *H*. *pilosella* (t-test, t = -4.16, p<0.001, Fig 4D). No significant difference in the total number of hairs per leaf was found for *E*. *vulgare* (t-test, t = 1.57, p = 0.146, Fig 4D). Pearson correlations of the leaf size and hair density displayed a negative linear relationship between leaf size and leaf hair density for both plant species (*H*. *pilosella*: R^2^ = -0.43, p<0.001, *E*. *vulgare*: R^2^ *=* -0.48, p<0.001, S6 Fig).

## Discussion

The diverse effects of UV-B radiation on numerous organisms are being increasingly recognized [9], but our current understanding of plant’s phenotypic responses to UV-B is largely limited to experimental studies manipulating UV-B exposure of plants in the field, greenhouses or growth chambers. Here, we combined historical herbarium specimens with novel remote sensing data to investigate the effects of UV-B radiation on leaf hair traits in two invasive species. Consistent with our hypotheses, we observed that foliar hairs grew about 25% longer for *H*. *pilosella* and 25% denser for *E*. *vulgare* in records from the Southern Hemisphere. This corresponded to increased UV-B stress levels due to approximately 30–35% higher radiation at the collection sites in the Southern Hemisphere compared to the Northern Hemisphere. The BRT analysis and regression models also identified UV-B exposures to be significantly influential for hair length of *H*. *pilosella* and for hair density of *E*. *vulgare* even after we accounted for other bioclimatic variables. However, when we focused on each hemisphere separately or controlled directly for the effect of hemisphere in the regression analysis, we found no relationship between hair traits and UV-B variables.

Therefore, we did not confirm findings of previous experimental studies (e.g. [10, 11, 14]) which suggested that foliar hairs may respond to higher UV-B intensities, presumably offering protection against detrimental levels of radiation and giving advantage to invasive species when colonizing new environments [50, 51]. The combination of a lack of relationship between hair traits and UV-B radiation within hemispheres (despite quite large ranges in UV-B values) and the large difference in hair traits between hemispheres suggests that there is little evidence for causal relationship between UV-B and hair traits in our data. We cannot completely rule out UV-B radiation as a possible driver because its importance was supported by previous experimental studies and because UV-B radiation was the only considered variable that differed substantially between the hemispheres, while bioclimatic conditions (e.g. temperature, precipitation) and other considered variables (herbivory damage, collection date) were at similar levels. However, given that that the history of the plant populations and the levels of UV-B to which they were exposed are confounded, alternative explanations not related to the different levels of UV-B radiation are likely to explain the observed differences in leaf hair traits.

First, genotypic differences between native and introduced ranges may represent a plausible alternative explanation. These potential genotypic differences may result from the source stock population of the New Zealand specimens, from subsequent hybridization, from genetic adaptation or from the survival of different genotypes in the invaded range. It is known that numerous alien plant species have been able to evolve rapidly in new environments and that rapid adaptation might be an important force in invasion ecology (e.g. [52]). Some studies have demonstrated that alien plants can be very different in their growth form and size from conspecifics in the native range, due to evolution of clines in traits as a response to differing environmental conditions in the alien range [53, 54, 55]. However, determining whether the detected differences in hair traits are due to phenotypic plasticity or due to evolutionary shifts is difficult. The herbarium records used here, unfortunately, do not include such level of taxonomic detail that would allow accounting for spatial distribution of different genotypes. Nevertheless, previous studies suggested that the population of *H*. *pilosella* in New Zealand may not have originated from only a small and geographically-limited sample (e.g. the UK as often assumed) that spread via asexual reproduction (i.e. apomixis). For example, Trewick *et al*. [38] suggest that the New Zealand population includes variable genotypes coming from different places in the UK and Central/Eastern Europe, and that the genotype may have further developed in the invaded range. Thus, although the UV-B hypothesis is supported by previous growth chamber experiments that show UV-B effects on samples with identical genotypes [14, 34], the genotypic difference may be a possible explanation of observed differences in leaf hair traits between native and invaded ranges. These past experiments suggest that, even if the observed differences in hair trait expressions are genetically-based, UV-B could be partly responsible, either through phenotypic plasticity or through being the driver behind this evolutionary adaptation in the first place. Proof of genetic adaptation could potentially be detected by observing changes of hair traits over time. While we tested this assumption (see S2 and S4 Figs), we were unable to draw a final conclusion due to the limited time-span covered by the specimens collected in the Southern Hemisphere. Both species were introduced to New Zealand and elsewhere in the mid-19^th^ century (first reports date back to 1878 for *H*. *pilosella* and 1870 for *E*. *vulgare*), yet the used herbarium records only date back to the 1940s when the spread of the study species had already begun [56]. This indicates that there has been a substantial time-lag between introduction and spread during which genetic selection and adaptation might have occurred [57].

Second, environmental factors such as microclimatic conditions or soil nutrient status may represent another alternative explanation of observed leaf hair traits. Although the collection areas from which the majority of the specimens originated are located at similar latitudes with comparable climate (Fig 1), it remains unknown under what local site conditions the plants have grown. For example, more hairs and smaller, thicker leaves may form as a response to increased photosynthetically active radiation (PAR). It is also possible that in New Zealand the studied plants are associated with more open environments, making it difficult to distinguishing between responses to increased PAR versus UV-B radiation on individual sites. Thereby, information which could help explain and interpret the observed differences, such as population structures, biomass, plant size, PAR or short-term variations in the local climate, is missing. While field studies containing such data exist for *H*. *pilosella* [42], these cover a much smaller portion of the distribution and have been conducted in recent years and do not necessarily reflect the conditions encountered by the specimens collected in earlier years.

Third, different levels of herbivory by insects or slugs may affect leaf hairiness as has been described in many species (e.g. [58]). Although data on the abundance of herbivores are typically not part of herbaria records, we have still accounted for the potential effect of herbivory by analyzing the percent of damage visible on herbarium specimens. We found no significant difference between the native and alien ranges (S3 Fig), but that does not necessarily mean that the levels of herbivory were the same throughout the past. While these results suggest that phenotypic plasticity in response to herbivory is likely not driving the observed differences, it does not exclude the possibility that increased investment in leaf hair traits may be a genetic adaptation to higher levels of herbivory. To fully resolve the role of herbivores in the expression of leaf hair traits would require experimental testing or field surveys on living plant material because the collection of herbarium specimens is likely biased (individuals with no or minimal damage are probably preferred for herbaria collections).

Specimens of both plant species collected in the Southern Hemisphere also revealed reduced leaf area (Fig 4D, S5 Fig), a commonly observed response to higher UV-B levels, since smaller and thicker leaves reduce detrimental effects of UV-B [3, 59, 60]. However, leaf area is a highly plastic trait [61] and may also respond to other local environmental conditions (e.g. drought, shade) which are unknown for the herbarium records used here. Therefore, the observed differences in leaf area cannot solely be attributed to UV-B radiation, especially in the case of *E*. *vulgare* for which none of the tested UV-B variables were identified as influential factors for leaf area in a BRT analysis (S1 Fig).

We also found significant negative relationship between leaf area and leaf hair density for both plant species (S6 Fig). This result supports Roy *et al*. [49] who suggested that hair density is a complex, non-independent trait which is influenced not only directly by the number of hairs initiated per leaf but also indirectly by leaf size. The decreasing density of hairs is consistent with the growth of epidermal cell size, rather than the number of cells, as the leaf size increases. This may imply that large leaves are less protected by hairs than small ones, which may be a trade-off between plant size and its protection against UV-B radiation and/or herbivore damage. However, while not investigated here, leaf hair length might also show responses to UV-B in *E*. *vulgare*. Since hairs of *E*. *vulgare* cannot be measured on herbarium records, more research using fresh plant material is needed to fully elucidate this question. Such studies should also investigate other traits such as stomata density, cuticle thickness or the production of secondary compounds that might be affected by UV-B.

In addition to the uncertainties in the herbaria records, certain limits arise from using global climate datasets, such as glUV. The dataset is based on satellite measurements taken during the last decade, while the specimens of both study species date back to the early 1800s in the Northern and the 1940s in the Southern Hemisphere. Although there is no global long-term measurement of UV-B radiation available, the intensities of UV-B radiation have likely changed over time, with decreasing levels of UV-B in the Northern and increasing levels of UV-B in the Southern Hemisphere [19]. Thus, we can assume that the early collected specimens were exposed to slightly different levels of UV-B radiation than captured by the used UV-B variables.

Historical herbaria and museum collections provide great potential for studies of global change [28, 29, 30]. Especially in the context of biological invasions, combining herbaria records and novel remote sensing data represents a unique opportunity for future research of UV-B and other climatic effects on plant morphology and distribution. Global analyses of trait data, such as those available in the TRY database [62], in combination with climate data may help uncover distributional patterns of other species traits and examine factors that affected the spread of invasive species over time [63]. Although our findings were inconclusive and the study species were not randomly sampled from among successful invaders, previous experimental studies imply that plants that are able to cope with UV-B radiation due to specific morphological or physiological traits may have an advantage for invading areas with higher UV-B irradiation. Future observational studies of leaf hair traits should focus on investigating the spatial distribution of different genotypes and incorporating detailed site specific information, in order to disentangle the genetic changes from phenotypic responses and thus better understand the importance of UV-B radiation as a potential factor determining plant invasion success.

## Supporting information

S1 Table Correlation matrix of considered UV-B and bioclimatic variables. Bolding denotes correlations above 0.7 or below -0.7 (Dormann *et al*., 2013). Notes: Annual Mean UV-B (UVB1), Mean UV-B of Highest Month (UVB3), Mean UV-B of Lowest Month (UVB4), Sum of UV-B Radiation of Highest Quarter (UVB5), Sum of UV-B Radiation of Lowest Quarter (UVB6), Annual Mean Temperature (BIO1), Maximum Temperature of Warmest Month (BIO5), Minimum Temperature of Coldest Month (BIO6), Mean Temperature of Warmest Quarter (BIO10), Mean Temperature of Coldest Quarter (BIO11), Annual Precipitation (BIO12), Precipitation of Wettest Month (BIO13), Precipitation of Driest Month (BIO14), Precipitation of Wettest Quarter (BIO16), Precipitation of Driest Quarter (BIO17), Altitude (alt).
(DOCX)

S1 Fig Most important UV-B/bioclimatic variables (predictors) to explain leaf area data (response) based on the Boosted Regression Tree (BRT) analysis. All variables having more than 0% relative influence are shown. Maximum Temperature of Warmest Month accounts for most variation in leaf area for *Hieracium pilosella* (a; rel. influence 26.5%). Precipitation of Wettest Quarter accounts for most variation in leaf area for *Echium vulgare* (b; rel. influence 71.6%). See main text for full list of variables used in the BRT analysis.
(TIF)

S2 Fig Relationship between Mean UV-B radiation of Highest Month and phenotypic expressions of foliar hair density in *Hieracium pilosella*. Records from the Northern Hemisphere are denoted by open circles, records from the Southern Hemisphere are denoted by black triangles. Lines with p-values represent fitted linear regression models. Vertical boxplots with p-values from t-tests show the differences in hair density between the hemispheres. Horizontal boxplots with p-values from t-tests compare the intensities of UV-B radiation between the hemispheres. Abbreviations: NH = Northern Hemisphere, SH = Southern Hemisphere.
(TIF)

S3 Fig **Analysis of herbivory damage on herbarium specimen leaves on (a) *Hieracium pilosella* and (b) *Echium vulgare* specimens.** P-values represent outcomes of t-tests. Abbreviations: NH = Northern Hemisphere, SH = Southern Hemisphere.
(TIF)

S4 Fig **Analysis of potential effects of collection year on (a) foliar hair length, (b) foliar hair density in *Hieracium pilosella* and (c) foliar hair density in *Echium vulgare*.** Records from the Northern Hemisphere are denoted by open circles, records from the Southern Hemisphere denoted by black triangles. Lines with p-values represent fitted linear regression models.
(TIF)

S5 Fig **Relationships between Maximum Temperatures of the Warmest Month and phenotypic expressions of leaf area in *Hieracium pilosella* (a) and Precipitation of the Wettest Quarter and leaf area in *Echium vulgare* (b).** Records from the Northern Hemisphere are denoted by open circles, records from the Southern Hemisphere are denoted by black triangles. Lines with p-values represent fitted linear regression models. Vertical boxplots with p-values from t-tests show the differences in leaf area between the hemispheres. Horizontal boxplots with p-values from t-tests compare the Maximum Temperatures of the Warmest Month (a) and Precipitation of the Wettest Quarter (b) between the hemispheres. Abbreviations: NH = Northern Hemisphere, SH = Southern Hemisphere.
(TIF)

S6 Fig **Relationship between leaf hair density and leaf area on (a) *Hieracium pilosella* and (b) *Echium vulgare*.** Records from the Northern Hemisphere are denoted by open circles, records from the Southern Hemisphere denoted by black triangles. Lines with p-values represent fitted linear regression models.
(TIF)
