# Supplementary material for: Effects of UV-B radiation on leaf hair traits of invasive plants—Combining historical herbarium records with novel remote sensing data
Source: PLoS One. 2017 Apr 17;12(4):e0175671. doi: 10.1371/journal.pone.0175671 (PMC5393584; doi:10.1371/journal.pone.0175671)
Supplement: S1 Table — Bolding denotes correlations above 0.7 or below -0.7 (Dormann et al., 2013). Notes: Annual Mean UV-B (UVB1), Mean UV-B of Highest Month (UVB3), Mean UV-B of Lowest Month (UVB4), Sum of UV-B Radiation of Highest Quarter (UVB5), Sum of UV-B Radiation of Lowest Quarter (UVB6), Annual Mean Temperature (BIO1), Maximum Temperature of Warmest Month (BIO5), Minimum Temperature of Coldest Month (BIO6), Mean Temperature of Warmest Quarter (BIO10), Mean Temperature of Coldest Quarter (BIO11), Annual Precipitation (BIO12), Precipitation of Wettest Month (BIO13), Precipitation of Driest Month (BIO14), Precipitation of Wettest Quarter (BIO16), Precipitation of Driest Quarter (BIO17), Altitude (alt). (DOCX) [file pone.0175671.s001.docx]

|  | UVB1 | UVB3 | UVB4 | UVB5 | UVB6 | BIO1 | BIO5 | BIO6 | BIO10 | BIO11 | BIO12 | BIO13 | BIO14 | BIO16 | BIO17 | alt |
| --- | --- | --- | --- | --- | --- | --- | --- | --- | --- | --- | --- | --- | --- | --- | --- | --- |
| UVB1 | 1 |  |  |  |  |  |  |  |  |  |  |  |  |  |  |  |
| UVB3 | **0.98** | 1 |  |  |  |  |  |  |  |  |  |  |  |  |  |  |
| UVB4 | **0.96** | **0.92** | 1 |  |  |  |  |  |  |  |  |  |  |  |  |  |
| UVB5 | **0.99** | **0.97** | **0.94** | 1 |  |  |  |  |  |  |  |  |  |  |  |  |
| UVB6 | **0.87** | **0.76** | **0.89** | **0.87** | 1 |  |  |  |  |  |  |  |  |  |  |  |
| BIO1 | 0.47 | 0.42 | 0.51 | 0.44 | 0.45 | 1 |  |  |  |  |  |  |  |  |  |  |
| BIO5 | -0.02 | -0.01 | -0.03 | -0.01 | -0.01 | 0.00 | 1 |  |  |  |  |  |  |  |  |  |
| BIO6 | 0.01 | -0.03 | 0.03 | 0.02 | 0.07 | 0.26 | **-0.84** | 1 |  |  |  |  |  |  |  |  |
| BIO10 | 0.15 | 0.16 | 0.16 | 0.13 | 0.11 | 0.23 | **0.90** | **-0.87** | 1 |  |  |  |  |  |  |  |
| BIO11 | 0.24 | 0.21 | 0.26 | 0.24 | 0.23 | 0.28 | **-0.74** | **0.74** | -0.62 | 1 |  |  |  |  |  |  |
| BIO12 | 0.44 | 0.47 | 0.45 | 0.45 | 0.32 | -0.16 | 0.00 | -0.19 | 0.05 | 0.18 | 1 |  |  |  |  |  |
| BIO13 | 0.41 | 0.43 | 0.43 | 0.43 | 0.34 | -0.17 | -0.01 | -0.18 | 0.04 | 0.19 | **0.98** | 1 |  |  |  |  |
| BIO14 | 0.45 | 0.49 | 0.46 | 0.46 | 0.33 | -0.15 | 0.00 | -0.20 | 0.06 | 0.17 | **0.98** | **0.94** | 1 |  |  |  |
| BIO16 | 0.40 | 0.42 | 0.42 | 0.42 | 0.33 | -0.18 | -0.01 | -0.18 | 0.04 | 0.18 | **0.98** | **0.99** | **0.95** | 1 |  |  |
| BIO17 | 0.45 | 0.49 | 0.45 | 0.46 | 0.30 | -0.14 | 0.00 | -0.19 | 0.06 | 0.16 | **0.98** | **0.95** | **0.99** | **0.95** | 1 |  |
| alt | 0.21 | 0.22 | 0.15 | 0.25 | 0.23 | -0.68 | 0.02 | -0.33 | -0.05 | -0.15 | 0.54 | 0.56 | 0.53 | 0.56 | 0.52 | 1 |
