# Supplementary figures and images for: Effects of UV-B radiation on leaf hair traits of invasive plants—Combining historical herbarium records with novel remote sensing data
Source: PLoS One. 2017 Apr 17;12(4):e0175671. doi: 10.1371/journal.pone.0175671 (PMC5393584; doi:10.1371/journal.pone.0175671)

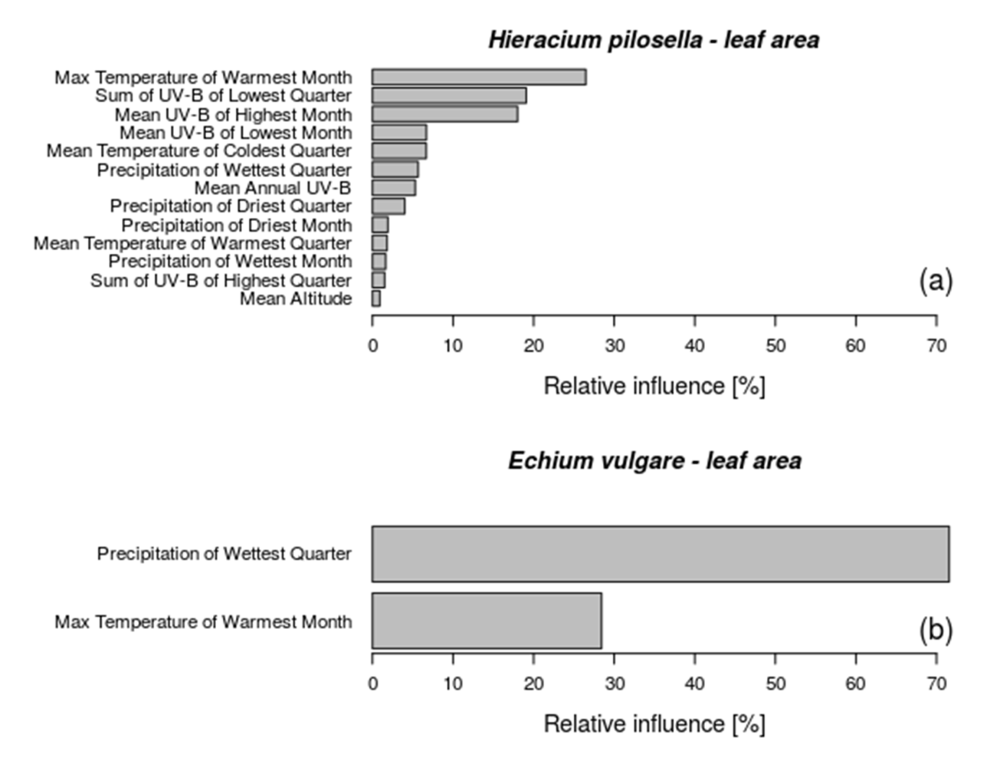

Supplement: S1 Fig — All variables having more than 0% relative influence are shown. Maximum Temperature of Warmest Month accounts for most variation in leaf area for Hieracium pilosella (a; rel. influence 26.5%). Precipitation of Wettest Quarter accounts for most variation in leaf area for Echium vulgare (b; rel. influence 71.6%). See main text for full list of variables used in the BRT analysis. (TIF) [file pone.0175671.s002.tif]

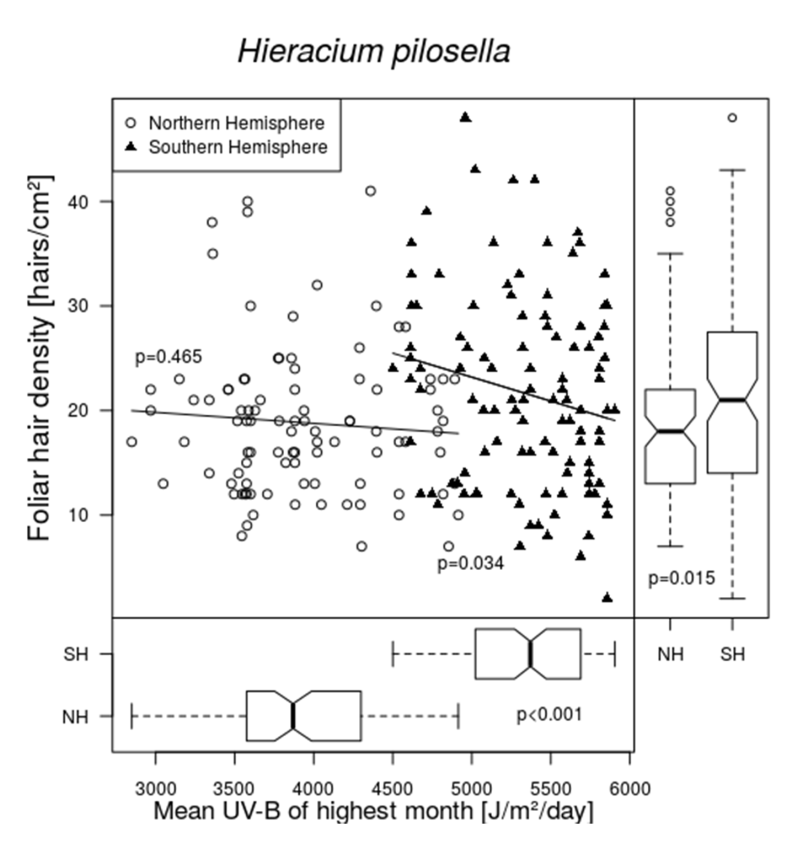

Supplement: S2 Fig — Records from the Northern Hemisphere are denoted by open circles, records from the Southern Hemisphere are denoted by black triangles. Lines with p-values represent fitted linear regression models. Vertical boxplots with p-values from t-tests show the differences in hair density between the hemispheres. Horizontal boxplots with p-values from t-tests compare the intensities of UV-B radiation between the hemispheres. Abbreviations: NH = Northern Hemisphere, SH = Southern Hemisphere. (TIF) [file pone.0175671.s003.tif]

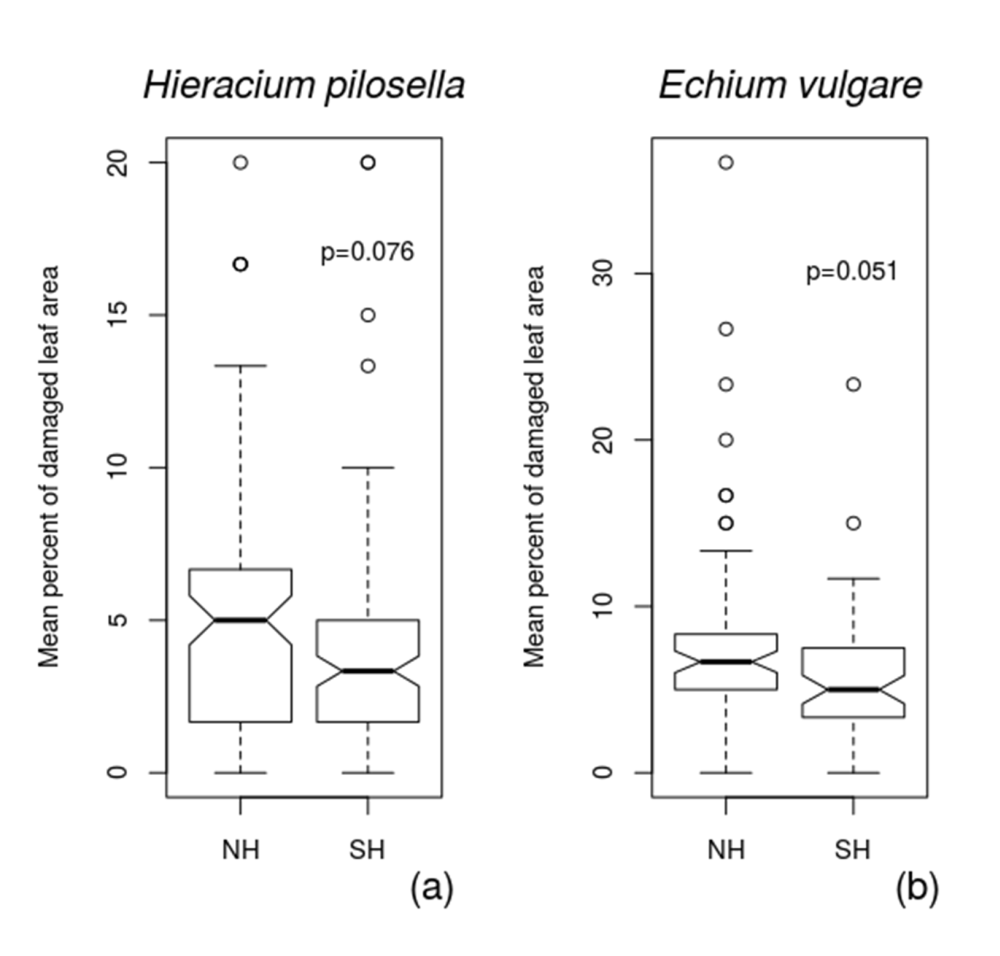

Supplement: S3 Fig — Analysis of herbivory damage on herbarium specimen leaves on (a) Hieracium pilosella and (b) Echium vulgare specimens. P-values represent outcomes of t-tests. Abbreviations: NH = Northern Hemisphere, SH = Southern Hemisphere. (TIF) [file pone.0175671.s004.tif]

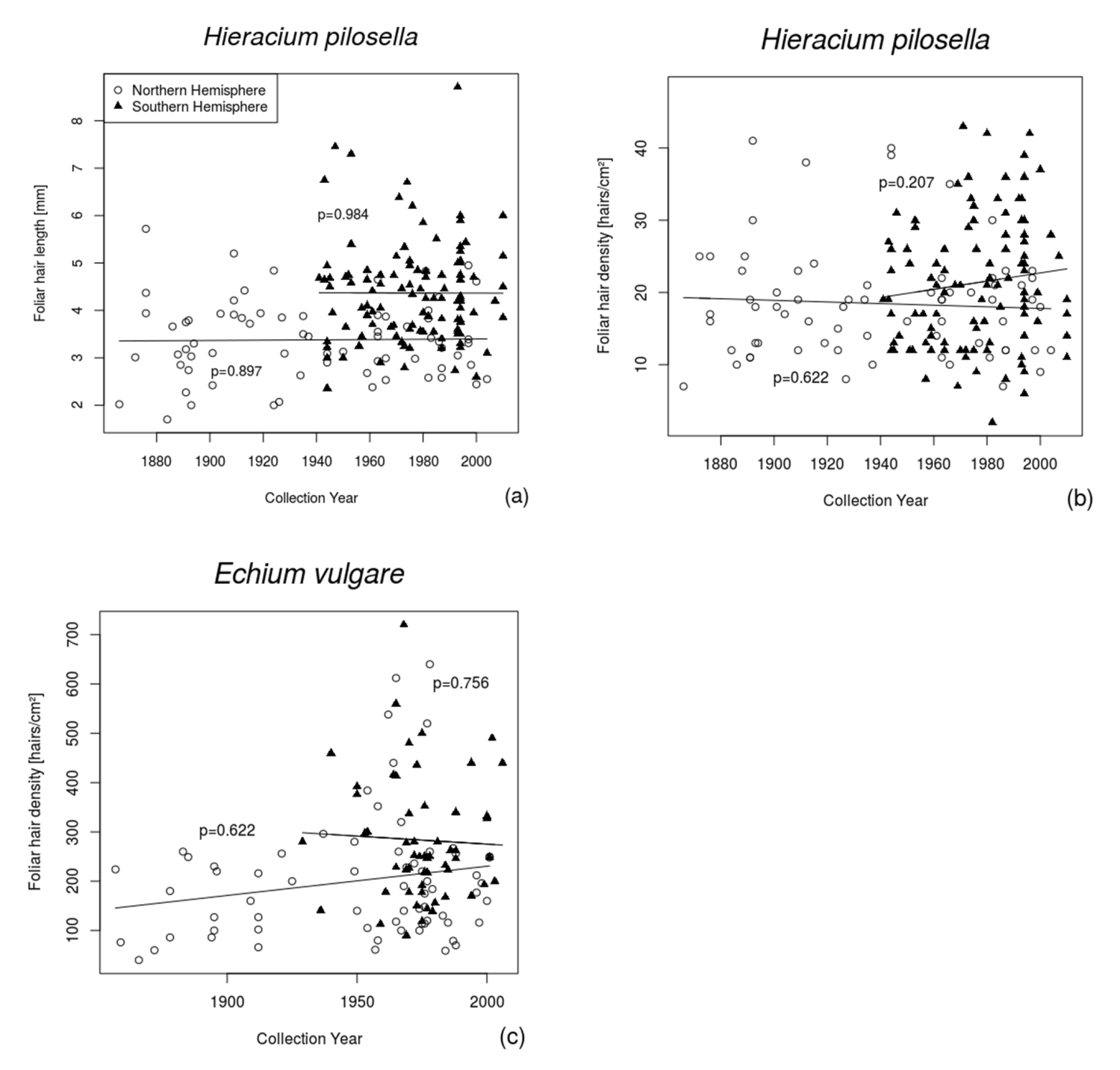

Supplement: S4 Fig — Analysis of potential effects of collection year on (a) foliar hair length, (b) foliar hair density in Hieracium pilosella and (c) foliar hair density in Echium vulgare. Records from the Northern Hemisphere are denoted by open circles, records from the Southern Hemisphere denoted by black triangles. Lines with p-values represent fitted linear regression models. (TIF) [file pone.0175671.s005.tif]

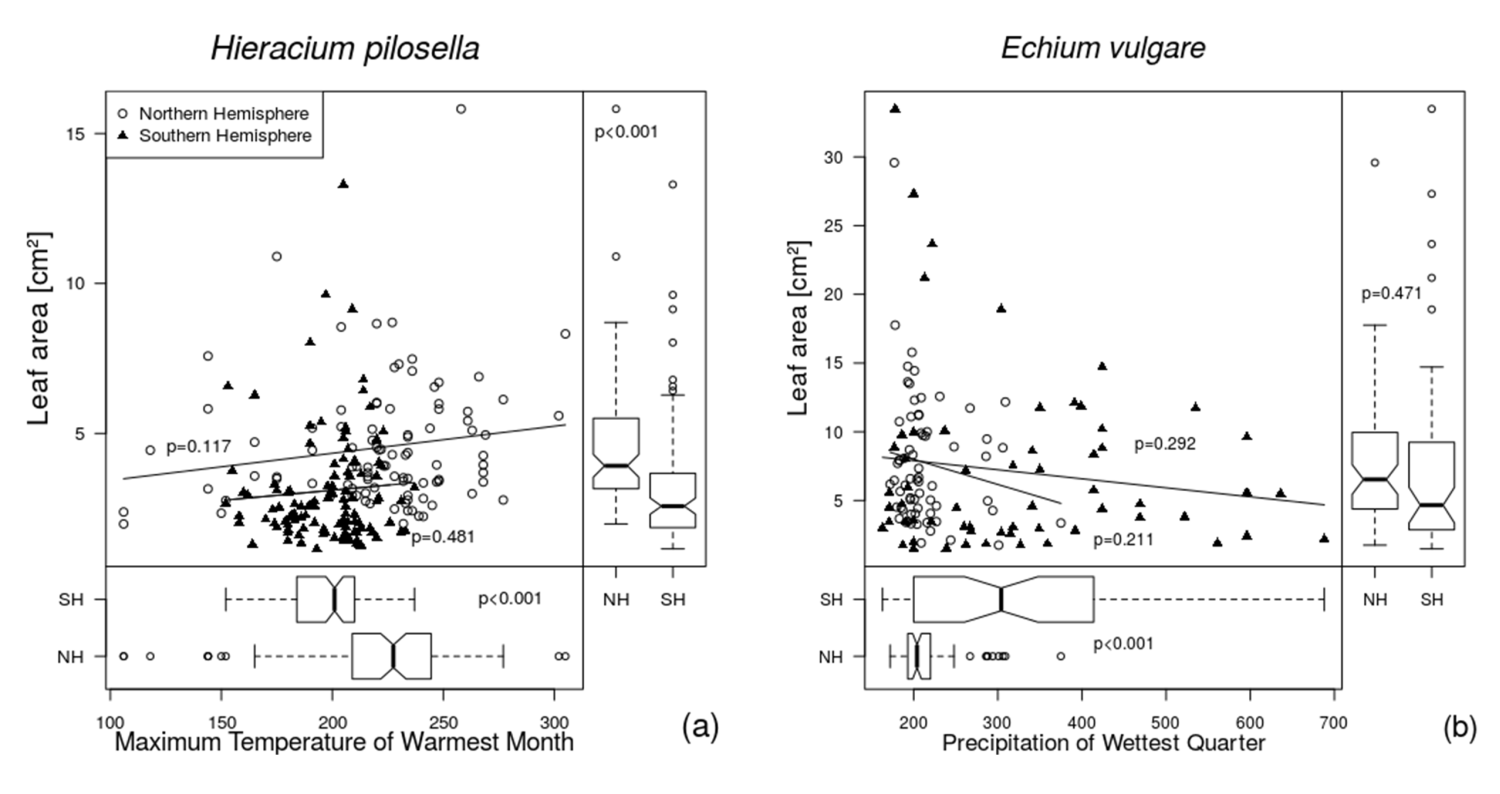

Supplement: S5 Fig — Relationships between Maximum Temperatures of the Warmest Month and phenotypic expressions of leaf area in Hieracium pilosella (a) and Precipitation of the Wettest Quarter and leaf area in Echium vulgare (b). Records from the Northern Hemisphere are denoted by open circles, records from the Southern Hemisphere are denoted by black triangles. Lines with p-values represent fitted linear regression models. Vertical boxplots with p-values from t-tests show the differences in leaf area between the hemispheres. Horizontal boxplots with p-values from t-tests compare the Maximum Temperatures of the Warmest Month (a) and Precipitation of the Wettest Quarter (b) between the hemispheres. Abbreviations: NH = Northern Hemisphere, SH = Southern Hemisphere. (TIF) [file pone.0175671.s006.tif]

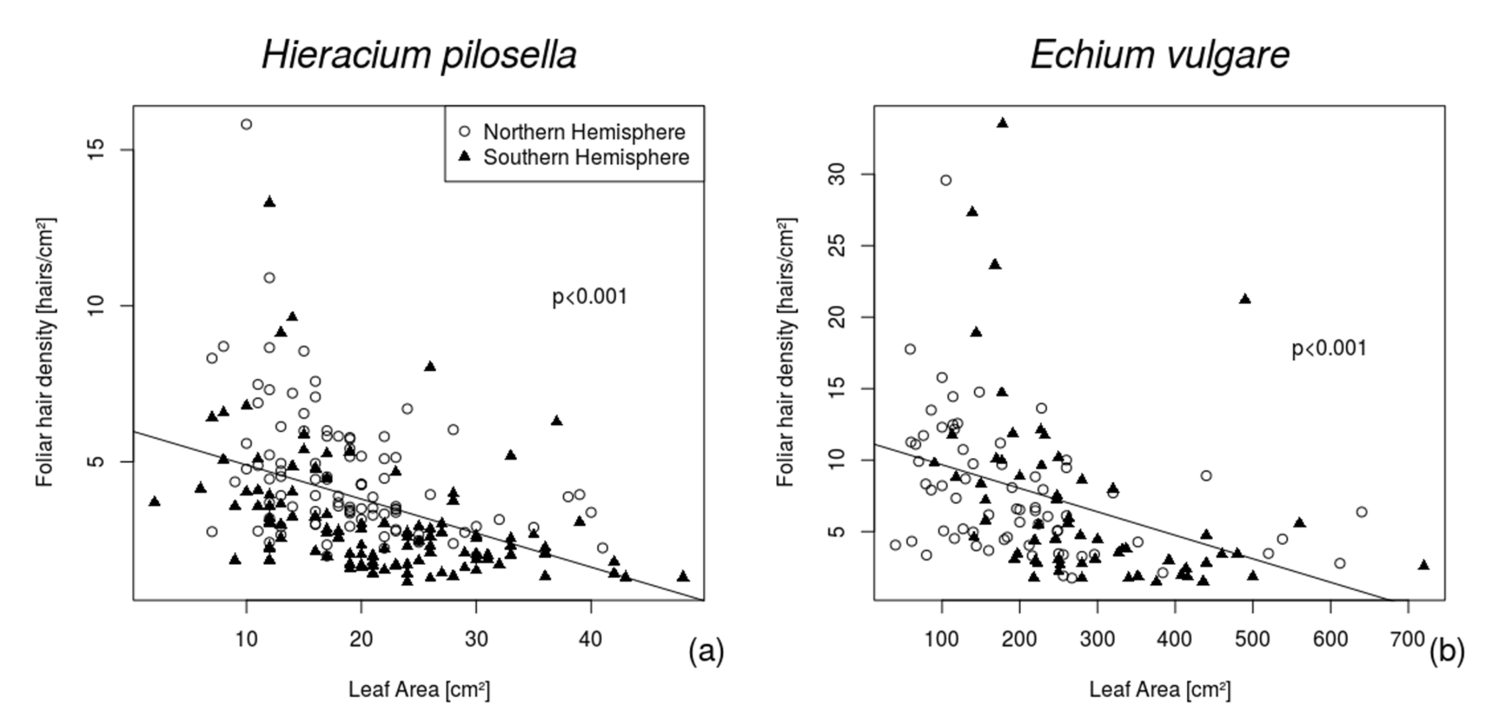

Supplement: S6 Fig — Relationship between leaf hair density and leaf area on (a) Hieracium pilosella and (b) Echium vulgare. Records from the Northern Hemisphere are denoted by open circles, records from the Southern Hemisphere denoted by black triangles. Lines with p-values represent fitted linear regression models. (TIF) [file pone.0175671.s007.tif]
